# Subunit Organisation of In Vitro Reconstituted HOPS and CORVET Multisubunit Membrane Tethering Complexes

**DOI:** 10.1371/journal.pone.0081534

**Published:** 2013-12-02

**Authors:** Zhong Guo, Wayne Johnston, Oleksiy Kovtun, Sergey Mureev, Cornelia Bröcker, Christian Ungermann, Kirill Alexandrov

**Affiliations:** 1 Department of Cell and Molecular Biology, Institute for Molecular Bioscience, The University of Queensland, Brisbane, Queensland, Australia; 2 Department of Biology/Chemisty, University of Osnabrück, Osnabrück, Germany; University of South Florida College of Medicine, United States of America

## Abstract

Biochemical and structural analysis of macromolecular protein assemblies remains challenging due to technical difficulties in recombinant expression, engineering and reconstitution of multisubunit complexes. Here we use a recently developed cell-free protein expression system based on the protozoan *Leishmania tarentolae* to produce *in vitro* all six subunits of the 600 kDa HOPS and CORVET membrane tethering complexes. We demonstrate that both subcomplexes and the entire HOPS complex can be reconstituted *in vitro* resulting in a comprehensive subunit interaction map. To our knowledge this is the largest eukaryotic protein complex *in vitro* reconstituted to date. Using the truncation and interaction analysis, we demonstrate that the complex is assembled through short hydrophobic sequences located in the C-terminus of the individual Vps subunits. Based on this data we propose a model of the HOPS and CORVET complex assembly that reconciles the available biochemical and structural data.

## Introduction

Small GTPases of the Rab and Arf families confer specificity and directionality to membrane trafficking steps such as vesicle budding, transport, tethering, docking, and fusion. They function by switching between active and inactive conformations in a spatially and temporally regulated manner. According to the current model, progression of the vesicular structure along the trafficking route is accompanied by sequential recruitment and subsequent release of specific Rab GTPases. Movement of cargo through the endocytic-lysosomal pathway is controlled by Rab5 and Rab7 GTPases, which define early and late endosomal compartments, respectively. Exchange of Rab5 for Rab7 is controlled by multiple factors including multisubunit tethering complexes (MTC) CORVET and HOPS that are critical for late endosomal and lysosomal biogenesis [1,2]. 

Both complexes share a four-subunit core (Vps11, Vps16, Vps18, and Vps33) and two additional, compartment-specific subunits. CORVET contains Vps3 and Vps8 subunits and interacts with Rab5 or its yeast orthologue Vps21 [2,3]. HOPS contains Vps39 and Vps41 subunits and interacts with Rab7 and its yeast Ypt7 GTPases [4] that operate at the late endosome to vacuole route [5]. CORVET is essential for traffic into late endosomes, while HOPS is involved in control of multiple steps of endocytic transport leading to lysosome. This includes transport of late endosomes, autophagosomes, and Golgi-derived AP-3 vesicles [6]. 

HOPS and CORVET belong to a large and structurally diverse family of tethering complexes that include TRAPP, COG, DSL, GARP and exocyst complexes ( for review 7). The shared features of these complexes are multisubunit architectures and the ability to interact with multiple small GTPases, in particular RabGTPases. The later interactions involve binding with the activated form of RabGTPase [8] or in the case of TRAPP complex, through guanine nucleotide exchange (GEF) activity [9]. 

Although the tethering complexes were discovered over a decade ago, progress in their analysis has been relatively slow. This is mainly due to their multisubunit architecture, which has precluded recombinant expression and hence detailed structural and biochemical analysis. The only notable exception is TRAPPI, which was reconstituted from recombinantly expressed subunits and subsequently crystallized [10]. In the case of other complexes, only structures of fragments of individual subunits or subcomplexes are known. Recently, several tethering complexes including COG, TRAPPII and HOPS were isolated from native sources by affinity purification and analyzed by electron microscopy [11-13]. 

HOPS and CORVET remain the least biochemically studied tethering complexes mainly due to the very large size of individual subunits, which preclude expression or reconstitution in prokaryotic expression systems. This is a shared problem in the analysis of macromolecular complexes where lack of an efficient recombinant expression route, and therefore ability to rapidly engineer these assemblies, presents an impediment to their analysis. So far, our knowledge of HOPS/CORVET assembly and interactions comes from two-hybrid system analysis, pull down experiments from yeast cells transformed with affinity tagged constructs, or analysis of individual subunits expressed in insect cells [14,15]. In addition, the overall arrangement of HOPS allowed the approximate localization of subunits in the EM structure, though their precise arrangement within the complex is not yet clear [16]. This is complicated by the fact that five (Vps11, 16, 18, 39, 41) of the six subunits have a predicted similar domain organization with an N-terminal β-propeller and a long α-solenoid domain at the C-terminal part [17]. Similar proteins like the COPII subunits have an extended structure [18] , which makes further predictions of the subunit arrangement in the absence of higher resolution structures within HOPS difficult. Only recently, the molecular interaction between Vps33 and parts of Vps16 have been described [19], though it is unclear, how these proteins assembly into the remaining HOPS or CORVET. The fact that both complexes are essential for yeast biogenesis complicates this analysis, which needs to be carried out on the background of an at least partially functional endocytic pathway. 

Biochemical and structural analysis of macromolecular protein assemblies is plagued by several interconnected problems. First, only a small fraction of eukaryotic protein complexes can be reconstituted from the individual expressed and purified subunits due to a co-translational or chaperon-assisted native assembly route [20]. These problems can be resolved by co-expression but simultaneous recombinant production of multiple subunits is far from trivial. In an oversimplification, the chances of expression of a subunit of a protein complex in functional form decreases with the power of the number of domains in this complex. Although exceptions are not uncommon, functional expression of large (>50 kDa) eukaryotic proteins typically requires eukaryotic translation and folding machinery. The second problem relates to versatility of the expression systems, as biochemical and structural analysis typically requires extensive engineering of the target protein(s). In the case of multiprotein complexes, this leads to geometric expansion in the number of subunit variants and their combinations, resulting in prohibitively slow experimental timelines and unacceptable costs. 

Both of these limitations can be theoretically resolved using *in vitro* expression systems where an unlimited number of genes can be co-expressed and the relative gene dosage can be precisely controlled. Further, several *in vitro* expression systems can be primed with linear non-clonal templates generated by PCR synthesis, thus lifting the requirements for cloning and sequencing. The main limitation of the cell-free systems so far was the high cost and low yields of eukaryotic system and poor performance of prokaryotic system in folding multisubunit eukaryotic proteins [21]. 

Here, we used a recently developed cell-free expression system based on the protozoan *Leishmania tarentolae* to recombinantly produce the subunits of HOPS and CORVET complexes and then reconstitute both complexes *in vitro*. We establish the hierarchy of complex assembly and revise the previously proposed interaction maps. We then used truncation analysis to identify the domains of subunits responsible for the complex assembly, and demonstrated that both complexes are held together by binding domains located in the C-terminus of individual subunits. 

## Results and Discussion

### In vitro expression of subunits in the HOPS and CORVET complex

**Figure 1:**
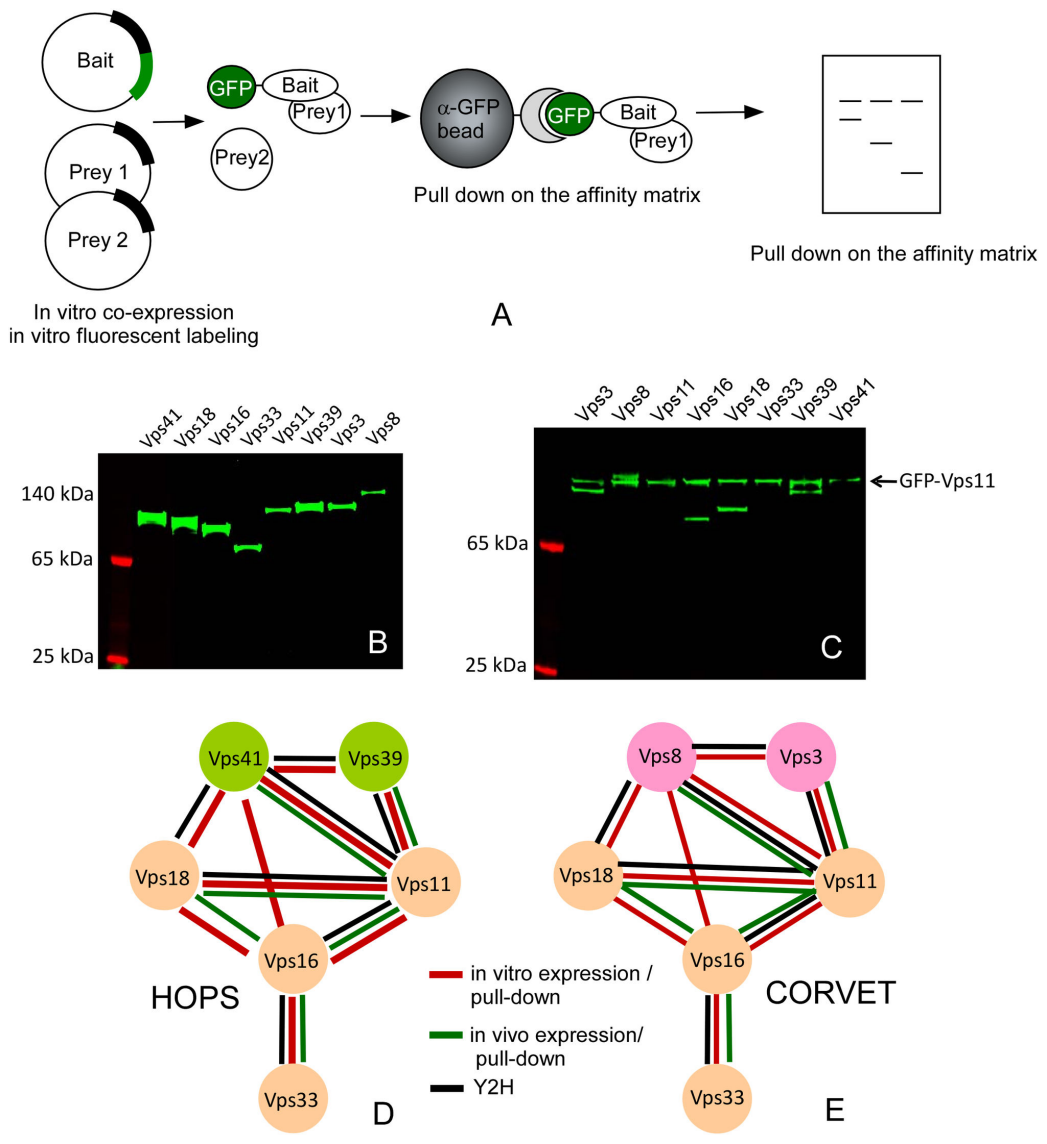
*In* *vitro* production of HOPS and CORVET subunits and pull down analysis of the subunit interactions. (A) Schematic representation of the experimental design. (B) SDS-PAGE analysis of the LTE lysate samples expressing individual subunits of the HOPS/CORVET complex. The samples were spiked with BODIPY-Lys-tRNA and the reaction products were visualized by scanning the unstained SDS-PAGE gels. (C) Interactions of Vps11 with other subunits of HOPS/CORVET complex. The GFP-tagged Vps11 was co-expressed with other subunits under conditions described in (B) and GFP-tagged proteins were isolated on the anti-GFP beads, eluted with SDS-PAGE loading buffer and resolved on the denaturing SDS-PAGE. The proteins were visualized by fluorescence scanning. (D) Interaction map of HOPS complex subunits based on the current study (red lines) and literature data (black and green lines). (E) the map of interactions in CORVET complex annotated as in D.

In order to analyze the architectures and structure of HOPS/CORVET complexes we attempted their reconstitution in the recently developed eukaryotic cell-free expression system based on the protozoan *L.tarentolae* [22]. This system has the advantage of high yield and relative ease of preparation, as it is based on a fermentable organism. We initially wanted to test whether the system would be able to produce large proteins efficiently. To this end, we PCR amplified the open reading frames coding for subunits of HOPS/CORVET complexes from *S.cerevisiae* genomic DNA and cloned them into pLTE vector [23]. In order to have a readily detectable and quantifiable signal for subunit expression, we also created N- or C-terminal EGFP fusion expression constructs for each subunit. The plasmids were translated in the LTE transcription/translation linked system [23] in the presence of BODIPY-Lys-tRNA. The reaction mixtures were resolved via SDS-PAGE gel. As can be seen in Figure 1B, all eight subunits were efficiently expressed as full-length products and samples contained little degradation/premature termination or unspecific products. 

### Co-expression and interaction analysis of HOPS/CORVET complex subunits

Next we wanted to establish whether the LTE system can be used for interaction analysis for the complex subunits. To this end, we took advantage of recombinant single chain camelid anti –GFP antibody crosslinked to the solid support. We previously demonstrated that this matrix can be used for one-step purification of EGFP tagged proteins from LTE extracts [24,25] . We iteratively co-expressed subunits of both complexes as untagged or C-/N-terminally EGFP tagged proteins in the presence of BODIPY-Lys-tRNA, and performed pulldown experiments on the GFP affinity matrix (Figure 1A). The matrix bound proteins were resolved by SDS-PAGE and visualized by fluorescent scanning. A fluorescent scan of typical interaction analysis is shown in Figure 1C, where EGFP-Vps11 was used as bait. This experiment demonstrates that Vps11 interacts with all HOPS/CORVET subunits except Vps33 and Vps41. We repeated the same experiment for all other subunits (Figure S1 in File S1). The resulting data is summarized in Figure 1D and E, and demonstrates that Vps11, 16 and 18 comprise the core of both complexes. Vps33 appears to be involved only in one interaction with Vps16, while the HOPS and CORVET specific subunits form extensive interactions with the core components. The obtained maps correspond in part with the maps established by other studies as outlined in Figures 1D and E. However, the analysis uncovered direct interactions among complex subunits undetected by the previous studies [14,15], as we found that Vps16 interacts directly with Vps41 and Vps8 in HOPS and CORVET complex respectively. Interaction of Vps16 with Vps41 is consistent with the recently published low resolution structure of HOPS complex [13]. 

### In vitro reconstitution of sub complexes and complete HOPS complex

**Figure 2:**
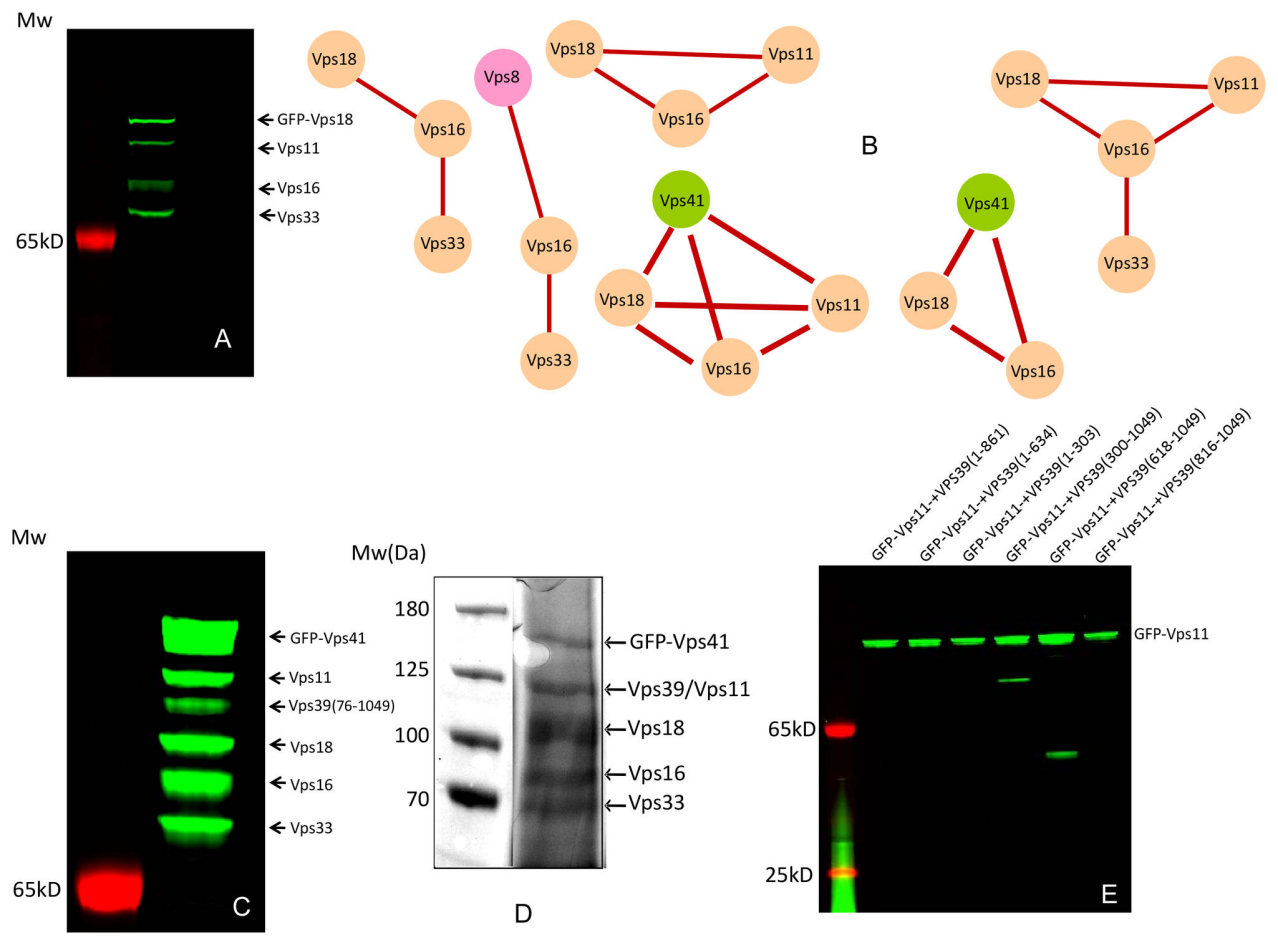
*In* *vitro* assembly of subcomplexes of HOPS and CORVET complexes. (A) An example of subcomplex containing Vps11, Vps16, GFP-Vps18 and Vps33 co-expressed in LTE extract and isolated on the anti-GFP matrix. (B) Graphic summary of subcomplexes isolated in this study. (C) Fluorescence scan of the SDS-PAGE gel loaded with *in* *vitro* reconstituted and affinity purified HOPS complex. The positions of the individual subunits are indicated on the right hand side. (D) Coomassie stained SDS-PAGE loaded with the HOPS containing fraction eluted from the gel filtration column. (E) Identification of the complex forming domains in the Vps subunits of HOPS and CORVET complex. GFP-tagged Vps11 was co-expressed with the truncated variants of Vps39 subunit. The samples were processed as in A.

Having established the basic interaction architecture of the HOPS/CORVET complexes we wanted to test whether the core subcomplexes or even entire complex can be assembled *in vitro* using the developed approach. We therefore used the developed interaction maps to co-translate the subunits of both complexes and isolate the interacting proteins by affinity purification. Figure 2A shows a representative experiment where the subcomplex of Vps18, 11, 16 and 33 was isolated using the GFP tag on the Vps18 subunit. Further experiments established isolation of stoichiometric complexes containing the following subunits: Vps8, Vps16 and Vps33; Vps11, Vps16 and Vps18; Vps16, Vps18 and Vps33; Vps16, Vps18 and Vps41; Vps11, Vps16 Vps18 and Vps33; Vps11, Vps16, Vps18 and Vps41 (Figure 2B and Figure S2 and S3 in File S1). Combined with the literature reporting isolation of Vps39:Vps11 and Vps39:Vps18 and Vps11 complexes [14,15] [26], these data indicate the modular assembly of the HOPS and CORVET complexes driven primarily by the interactions of the individual subunits.

Next, we wanted to establish whether a complete HOPS/CORVET complex can be reconstituted *in vitro* and purified. We attempted reconstitution using two approaches. First, the individual subunits were co-translated using the above described plasmids. Second, the subunits were expressed individually and the resulting lysates mixed. Iteratively including subunits with GFP tags on N- or C-terminus we experimentally established that the best results were obtained when Vps41 was N-terminally tagged with GFP (Figure 2C). As Vps11 and Vps39 migrate at the same position on the SDS-PAGE, we used a truncated version of Vps39 (76-1049AA) that migrates lower than Vps11. Interestingly, previously described C-terminal tagging of Vps33 subunit did not lead to a successful complex purification in our set-up. To our surprise co-translation was not strictly required for complex formation, and the entire HOPS complex could be assembled by mixing the lysates expressing individual subunits. This is consistent with the functional assembly of two subcomplexes into functional HOPS complex [14]. However, the yields of the final complex were higher in the case of co-expression of HOPS subunits. The fact that the HOPS complex could be efficiently reassembled from the individual subunits despite their large size is intriguing. It may imply an autonomous nature of Vps subunits, and possibly indicate the dynamic structure of the complex. An alternative explanation is that the LTE lysate possesses significant chaperon activity and is able to prevent the aggregation of even very large heterologous proteins. 

Finally, we wanted to test whether *in vitro* reconstituted HOPS complex can be isolated in amounts sufficient for biochemical and structural analysis. We established a two-step purification approach that combines the Ni-NTA affinity chromatography of his-tagged complex followed by size exclusion chromatography. We co-translated the DNA templates containing His-GFP-Vps41, Vps11, Vps16, Vps18, Vps33 and full-length Vps39. After loading the translation mixture onto the Ni-column, the HOPS was eluted with buffer containing 250mM imidazole. The fractions containing GFP fluorescence were pooled and loaded onto Superdex G200 10/300GL column. The complex eluted at the expected position corresponding to 700 kDa (Figure S4 in File S1). SDS-PAGE analysis of fractions containing GFP fluorescence demonstrated co-elution of all six subunits. As expected, Vps11 and Vps39 were not resolved on the used SDS-PAGE system and appeared as a single band (Figure 2D). We typically obtained 15 µg of HOPS complex per ml of LTE lysate. Although the obtained amounts are insufficient for crystallographic analysis, they are suitable for future electron microscopy and biochemical characterization. 

**Figure 3:**
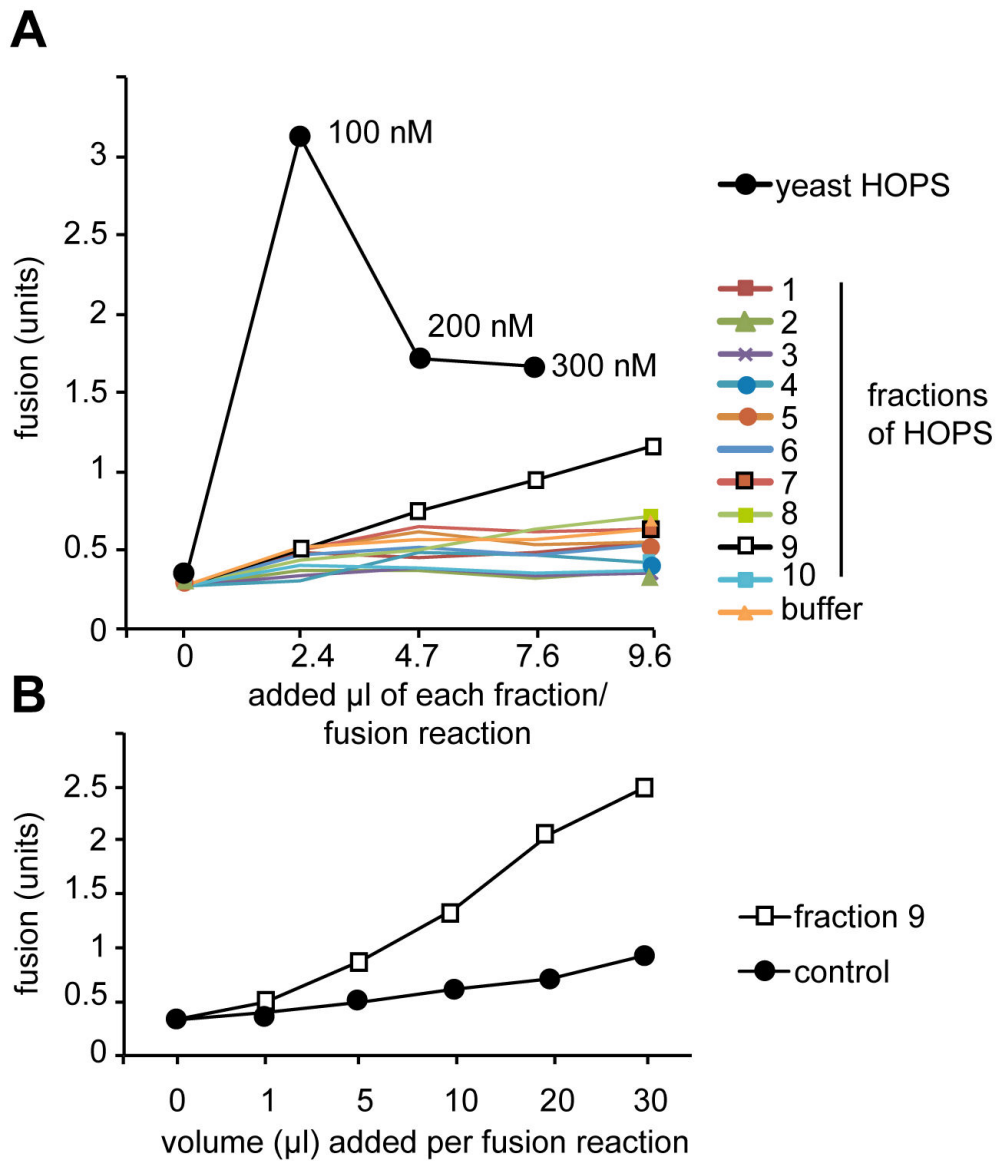
Recombinant HOPS can support vacuole fusion. Vacuoles were purified from each of the two tester strain (*pep4*∆ and *pho8*∆) that each carry the *vps11-1* mutation (Stroupe [29]and Wickner, 2006). Fusion reactions (30 µl total) contained 3 µg of each vacuole type in fusion salt (0.5 mM MgCl_2_, 125 mM KCl, 20 mM PIPES/KOH, pH 6.8, 200 mM sorbitol) and an ATP-regenerating system [14]. (A) The indicated volumes of each fraction of the HOPS purification were titrated into the fusion reaction. Reactions were incubated for 90 min at 26°C, and then assayed for Pho8 activity by addition of p-Nitrophenolphosphate [14]. As a control, purified yeast HOPS complex [16] was added at the indicated concentrations. (B) Titration of fraction 9 at higher volumes into the fusion reaction. The analysis was conducted as in (A). Control indicates the addition of volumes of purification buffer into the reaction.

To test for functionality of the reconstituted complex, we used the previously established vacuole fusion assay [27]. For this assay, vacuoles are purified from two tester strains, of which one lacks the major protease Pep4, but contains unprocessed alkaline phosphatase, whereas the other lacks the same enzyme and contains Pep4. Upon fusion of both vacuole type and luminal mixing, Pep4 cleaves the propetide from immature alkaline phosphatase (proPho8) and converts it to the active enzyme (Pho8). Cleavage of p-nitrophenol-phosphate to yellow p-nitrophenol provides a convenient assay of successful fusion (described in [28]). Unlike the original vacuole fusion, we used here vacuoles that contained a *vps11-1* mutation, which makes them dependent on purified HOPS [14] [29]. We then added fractions of the purification to the assay to test for HOPS activity. In comparison to wild-type HOPS, which had optimal activity at around 100 nM, we obtained only background activity for most fractions except fraction 9 (Figure 3A). When we increased the volume in the fusion assay, we detected robust activity above background, indicating that our preparation is functional in the fusion assay (Figure 3B). 

### Identification of complex assembly domains in Vps subunits

We next wanted to establish which domains of Vps subunits are responsible for the assembly of HOPS and CORVET complexes. To perform the saturating unbiased mapping of domains mediating complex assemblies, we chose to use a cloning-free approach for the generation and rapid interaction analysis of subunit fragments. In this approach the fragments of Vps subunits were amplified by PCR and tagged with T7 promoter and translation initiation sequences using an overlap extension PCR [23,24]. These allowed us to generate on average 6 truncations per subunit. The linear templates coding for fragments of subunits were co-translated in the presence of the individual EGFP tagged bait subunits and the resulted translation products were subjected to precipitation on the anti-GFP matrix and visualization on the SDS-PAGE as described above. The outcome of a representative experiment is shown in Figure 2E, where truncation analysis of Vps 39 identified the binding site for Vps11 between residues 618 and 816 of the former. The identified binding site is close but not identical to the previously identified binding site located between residues 901 and 974 of Vps39 [14]. Systematic analysis of the interaction domains revealed that Vps subunits assemble via on average 300 amino acids long interacting domains located on the C-terminus of individual subunits (Table 1). 

**Table 1:** Segments of Vps subunits mediating assembly of CORVET and HOPS complexes.

**GFP-Vps3**	**GFP-Vps11**	**GFP-Vps16**	**GFP-Vps18**	**GFP-Vps39**	**GFP-Vps41**
	Vps16(479-798)	Vps8(950-1274)	Vps8(950-1274)		
Vps11(737-1029)	Vps18(733-918)	Vps18(733-918)	Vps11(737-1029)	Vps11(737-1029)	Vps16(479-798) Vps18(733-918)
	Vps39(618-1049)	Vps33(440-691)	Vps16(479-798)		

Interestingly, it appeared from the analysis that the same domain can be involved in formation of complexes with different subunits. For instance, the fragment of Vps11 containing residues 737-1029 interacts with Vps3, Vps18 and Vps 39, while Vps16 uses fragment 479-798 to associate with Vps18 and Vps11 (Table 1). 

**Figure 4:**
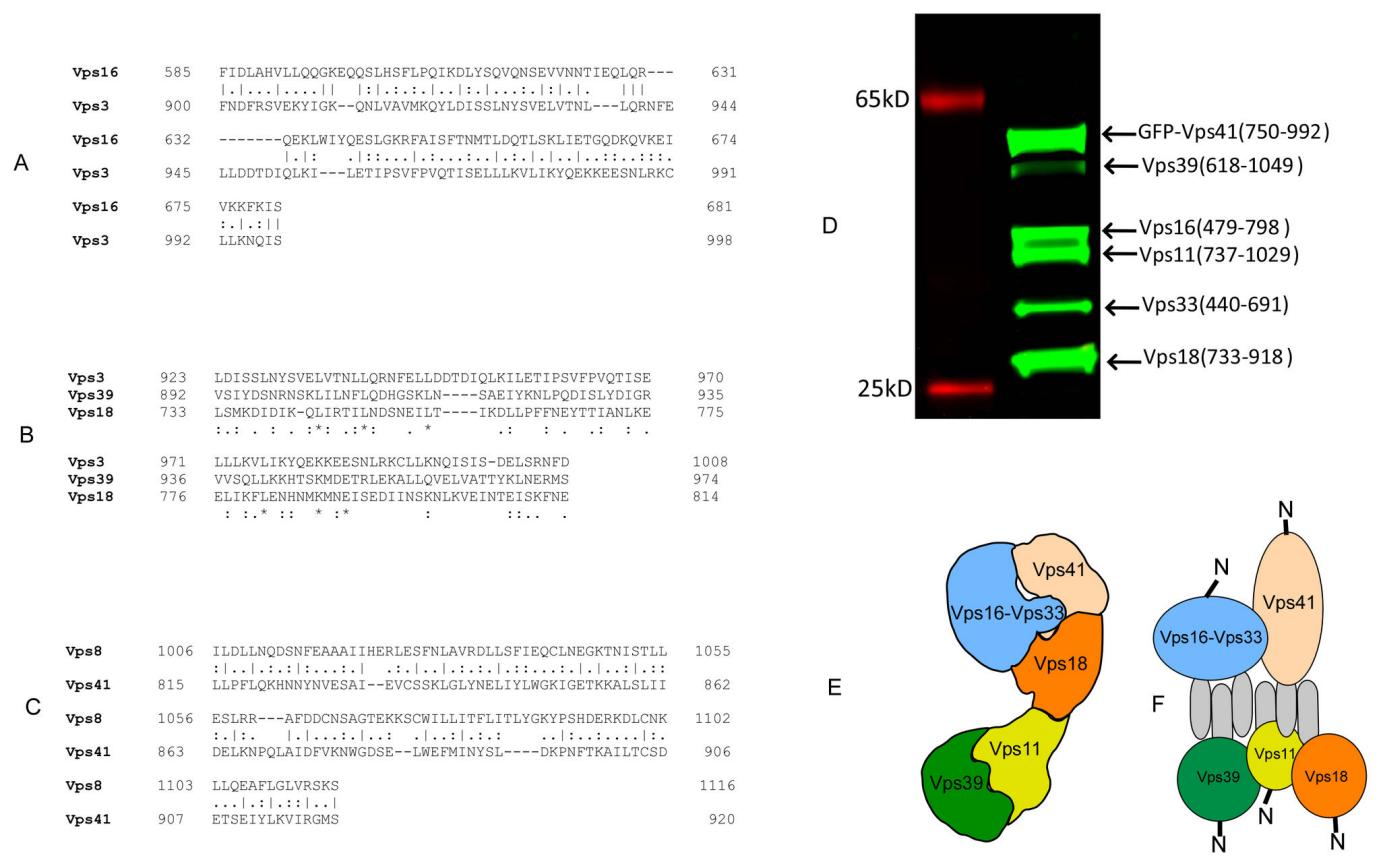
Analysis of HOPS/CORVET complex assembly domains (A) sequence alignment of VPS11 interacting domains of Vps3 and Vps16. (B) Multiple sequence analysis of the complex assembly domains of Vps3, Vps16 and Vps39. (C) Sequence alignment of the Vps8 and Vps41 interacting domains. (D) Reconstitution of the interaction core of HOPS complex. The truncated subunits we co-expressed with GFP-tagged Vps41 subunit and isolated on the anti-GFP matrix as described above. (E) Organization of HOPS complex based on the low resolution structure of yeast produced complex [13]. (F) Proposed mode of complex assembly based of the EM reconstruction and the presented biochemical results.

Having identified the interacting regions in Vps subunits we searched for information about their possible mode of interaction. Sequence alignments demonstrated that many of the identified segments contain regions of significant homology. The identified homology sequences fall into two groups: one formed by Vps3, Vps16, Vps18 and Vps39, and the other containing Vps8 and Vps41 [15]. While interacting segments of all four former proteins could not be aligned satisfactorily, pair wise alignments and the alignment of Vps3, Vps18 and Vps39 revealed a conserved signature of hydrophobic amino acids enriched in leucine and isoleucine (Figures 4A and B). Remarkably, although Vps8 and Vps41 do not display obvious homology to the former group the homology region between these proteins is also enriched in hydrophobic amino acid residues (Figure 4C). 

We next tested whether the identified interacting regions are solely sufficient for assembly of the HOPS complex. We thus performed co-expression of truncated subunits of HOPS in the presence of the GFP tagged Vps41 fragment (750-992). As shown in Figure 4D, all 5 truncated subunits could be isolated by a pull-down on the anti-GFP matrix, indicating that the truncated subunits could still assemble into the high affinity complex and that the identified regions constitute the primary determinants of complex assembly. This is a very interesting observation in the context of the recently published low-resolution structure of HOPS complex that revealed an extended assembly with bulbs on both ends (Figure 4E). According to this model several subunits such as Vps41 and Vps11 or Vps41 and Vps39 that had been shown to biochemically interact by this and other studies are located at the distant ends of the assembly. One possible explanation of this apparently contradictory result is parallel or antiparallel bundle of linear C-terminal interacting regions along the length of the complex (Figure 4F). The modest length of the interaction segments and the presence of the conserved hydrophobic amino acid residues may indicate assembly of a six-strand bundle with a hydrophobic core, which is possibly too small to be resolved by the EM analysis as an independent domain. In agreement, we recently showed that CORVET assembled if both N-terminal domains of Vps3 and Vps8 were deleted, whereas C-terminal deletions resulted in the disassembly of CORVET [30]. Crystallographic analysis of the identified core HOPS complex (ca. 180kDa) should address this question in future studies. 

In summary, we demonstrate that multiprotein assemblies composed from subunits as large as 140 kDa can be recombinantly produced in functional form in the LTE system. To our knowledge this represents the largest eukaryotic protein complex reconstituted *in vitro*. The flexibility of the LTE system combined with relatively high yield and ability to produce large eukaryotic proteins enabled us to perform the above-described interactions within six weeks. This represents a major improvement in the ability to analyze complex protein assemblies as the HOPS complex cannot be produced in bacteria. Our data highlight the importance of the C-terminal domains of five HOPS and CORVET proteins in assembling an apparent core of the complex. Moreover, we identified novel direct interactions between Vps8 and Vps41 subunits with Vps16. Our data is consistent with recent studies on the human HOPS subunits, where the C-terminal domains of Vps11, 16, 18, 39 and 41 were identified as the major binary interaction modules [19]. While the interactions demonstrate high specificity under the chosen experimental conditions, it is noteworthy that Leishmania extract may also contains Leishmania homologues of Vps proteins. Although large evolutionary distance between species makes cross reactivity of endogenous and recombinant Vps proteins is unlikely, it cannot be formally excluded. 

The combination of truncation and interaction analysis has revealed that the complexes assemble through interaction of relatively short stretches located in the C-terminal domains of the subunits that share conserved hydrophobic motifs. The developed technology and the presented data provide a new avenue to for detailed structural analysis of HOPS/CORVET complexes as well as other multiprotein assemblies. 

## Experimental Procedures

### LTE lysate preparation

The LTE lysate was obtained from the University of Queensland Protein Expression Facility http://uq.edu.au/pef/4pages/invitro.htm. The extracts were prepared as described elsewhere [23]. 

### Template Preparation, In Vitro Translation and Pull down Analysis

Template preparation was carried out by either cloning the full-length genes into pLTE vector containing the Species-Independent Translational Sequences (SITS) [22,23], or by amplifying the gene fragments and fusing them to SITS coding sequence by overlap extension PCR [24]. 

For the *in vitro* translation, the mixture of DNA template and LTE was incubated at 27 °C for 2.5 hours as described [23]. The reactions were supplemented with 1:500 dilution of BODIPY-Lys-tRNA (Promega) to achieve labeling of the resulting proteins. Typically for pull-down analysis 10 nM of plasmids coding for bait and prey proteins were co-translated in 100 µl LTE reaction. In case of PCR –generated templates and the reactions were primed with EtOH precipitated PCR product from 100 µl PCR reaction. For affinity isolation the translation mixtures were incubated with 20 µl 50%(V/V) GFP-Cap beads with gentle agitation for 15 min. Subsequently, the beads were washed five times with 250 µl buffer containing 50 mM NaHPO_4_ pH 8, 300 mM NaCl, 0.1% Triton. Finally, 20 µl of pre-warmed SDS-PAGE sample loading buffer was added to beads, and the samples were loaded onto SDS-PAGE and analyzed by fluorescence scanning using Typhoon Scanner (GE Healthcare) using 488 nm excitation laser and 520 BP40 emission filter.

To reconstitute the sub complexes, complete HOPS complex, total 20 nM of plasmids mixture were translated and processed as described above. 

### Preparative expression and purification of HOPS complex

To isolate the reconstituted HOPS complex, a plasmid mixture containing 6nM of 6His-GFP-Vps41, and 3 nM of Vps11, Vps16, Vps18, Vps33 and Vps39, was co-translated in 30 ml LTE at 27 °C for 2.5 hours. The crude mixture was loaded onto 1 ml Ni-NTA HiTrap column (GE healthcare) and washed with the buffer containing 50 mM NaHPO_4_ pH8, 300 mM NaCl, 20 mM imidazole. The complex was eluted with the buffer containing 50 mM NaHPO_4_, pH 8, 300 mM NaCl and 250 mM imidazole. The fractions containing GFP fluorescence were pooled and concentrated to 0.5 ml using Amicon Ultra Centrifugal Unit (Millipore). Finally, the sample was loaded onto Superdex G200 10/300GL column (GE healthcare) equilibrated with the buffer containing 50 mM Tris-HCl, pH 8, 200 mM NaCl, and 1 mM TECP. The chromatography was driven by the Akta Prime FPLC Protein Purification (GE healthcare) with the flow rate of 0.5 ml/min. The fractions containing GFP fluorescence were analyzed on 12.4% SDS-PAGE.

## Supporting Information

File S1 Figure S1, Pull down analysis of HOPS/CORVET complex subunit interactions. Figure S2, Identification of the complex-forming domains in Vps subunits of HOPS and CORVET complexes. Figure S3, In vitro assembly of subcomplexes of HOPS and CORVET complexes.
(DOCX)
