# Correction: Subunit Organisation of In Vitro Reconstituted HOPS and CORVET Multisubunit Membrane Tethering Complexes

**DOI:** 10.1371/annotation/3b445e95-4f8c-4238-9895-e05f49949f20

**Published:** 2014-01-02

**Authors:** Zhong Guo, Wayne Johnston, Oleksiy Kovtun, Sergey Mureev, Cornelia Bröcker, Christian Ungermann, Kirill Alexandrov

A funding organization and grant were incorrectly omitted from the Funding Statement. The Funding Statement should read: "This work was supported by the Deutsche Forschungsgemeinschaft UN111/5-2; SFB 944) to C.U., and Australian Research Council grant DP1094080, Australian Research Council Future Fellowship FT0991611, and National Health Medical Research Council project grant 569652 and Program Grant 1037320 to KA. The funders had no role in study design, data collection and analysis, decision to publish, or preparation of the manuscript. 

