# Supplementary material for: Subunit Organisation of In Vitro Reconstituted HOPS and CORVET Multisubunit Membrane Tethering Complexes
Source: PLoS One. 2013 Dec 2;8(12):e81534. doi: 10.1371/journal.pone.0081534 (PMC3846719; doi:10.1371/journal.pone.0081534)
Supplement: File S1 — Figure S1, Pull down analysis of HOPS/CORVET complex subunit interactions. Figure S2, Identification of the complex-forming domains in Vps subunits of HOPS and CORVET complexes. Figure S3, In vitro assembly of subcomplexes of HOPS and CORVET complexes. (DOCX) [file pone.0081534.s001.docx]

**Supporting Information**

| **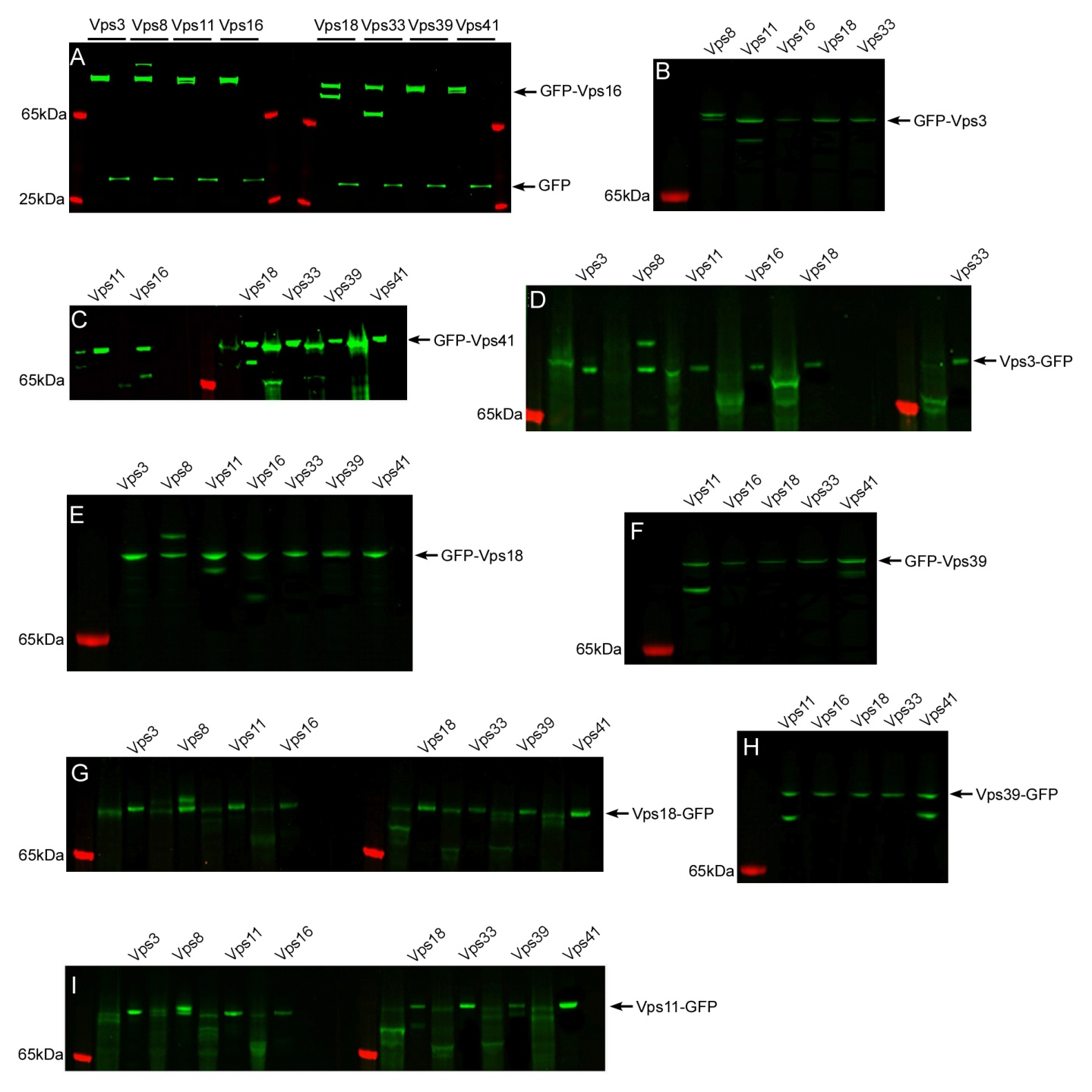** |
| --- |
| Figure S1: Pull down analysis of **HOPS/CORVET complex** subunit interactions. In the analysis the GFP tagged Vps subunits were co-expressed with other subunits. The samples were processed as in Figure 1C. In Figure A, negative controls were performed by co-expressing GFP with the Vps subunits. |

| **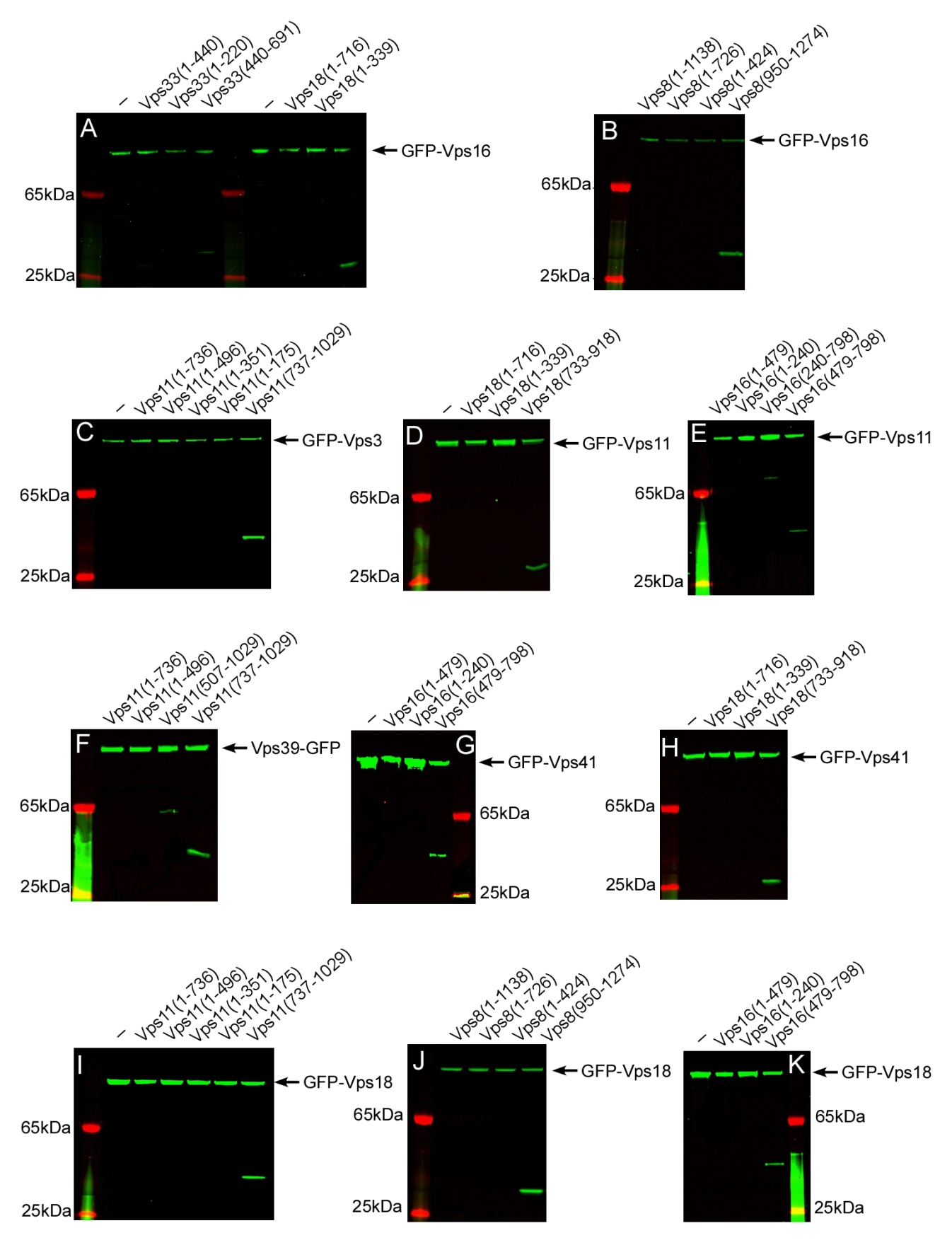** |
| --- |
| Figure S2: Identification of the complex-forming domains in Vps subunits of HOPS and CORVET complexes. (A-K) GFP tagged Vps protein was co-expressed with the truncated variants of its interacting subunit. The samples were processed as in Figure 2E. |

| **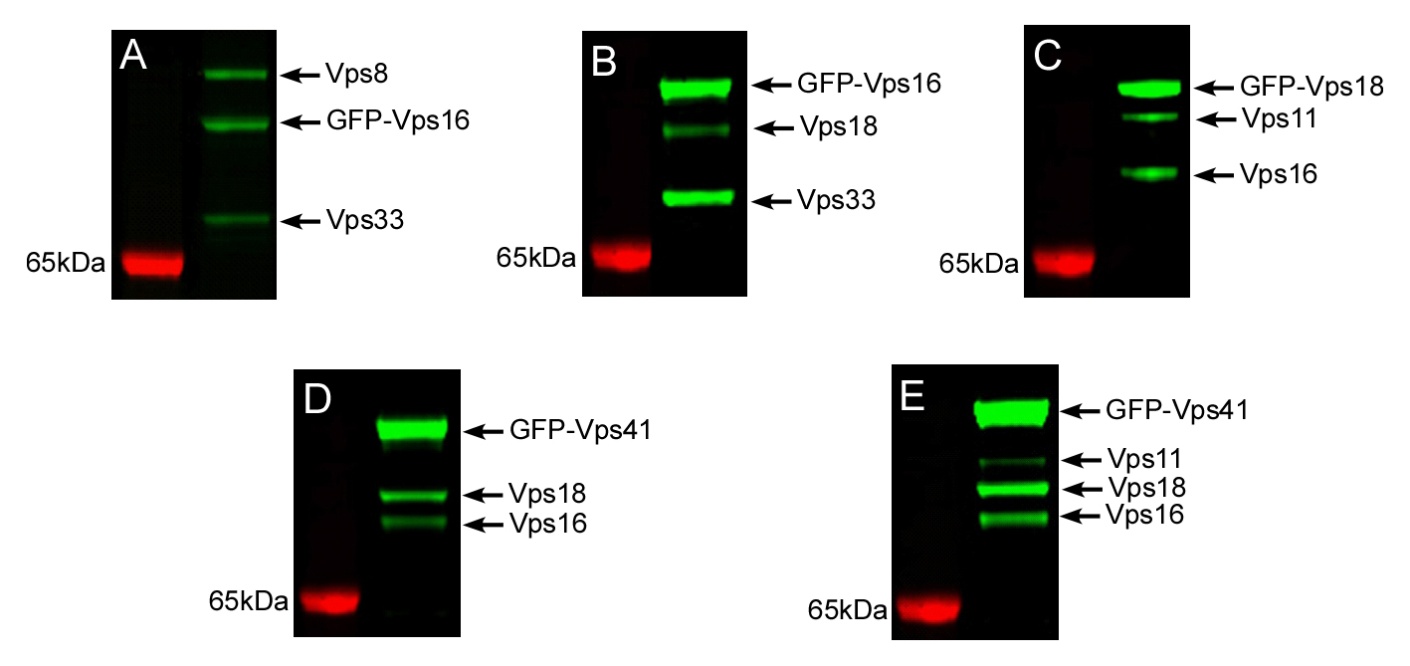** |
| --- |
| Figure S3: *In vitro* assembly of subcomplexes of HOPS and CORVET complexes (A-E). Subcomplex containing the Vps proteins co-expressed in LTE extract and isolated on the anti-GFP matrix. The samples were processed as in Figure 2A. |

| **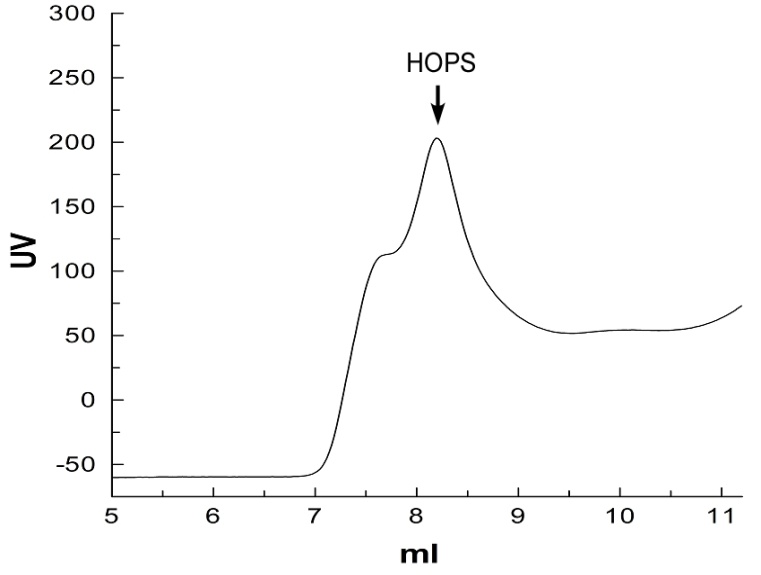** |
| --- |
| Figure S4: Purification of HOPS complex based on LTE extract. HOPS complex containing His-GFP-Vps41, Vps11, Vps16, Vps18, Vps33 and Vps39 was co-expressed in LTE extract and isolated preliminarily by Ni-matrix. The crude purified HOPS complex was further purified by Superdex 200 10/300GL column. The fractions containing HOPS associated fluorescence and HOPS subunits are indicated by an arrow. The elution volume for HOPS complex corresponds to 700 kDa protein. |
